# Pressure-Enabled Drug Delivery Approach in the Pancreas with Retrograde Venous Infusion of Lipiodol with Ex Vivo Analysis

**DOI:** 10.1007/s00270-020-02625-z

**Published:** 2020-09-07

**Authors:** Aravind Arepally, James Chomas, Steven C. Katz, David Jaroch, K. Pallav Kolli, Ethan Prince, Robert P. Liddell

**Affiliations:** 1 Division of Interventional Radiology, Piedmont Radiology, Piedmont Healthcare, 1984 Peachtree Road, Suite 505, Atlanta, Georgia 30309 USA; 2 Formerly TriSalus Life Sciences, Inc, Westminster, USA; 3 Office of Therapeutic Development and Department of Surgery, Roger Williams Medical Center, Providence, USA; 4 Department of Surgery, Boston University School of Medicine, Boston, USA; 5 TriSalus Life Sciences, Inc, Westminster, USA; 6 Department of Radiology and Biomedical Imaging, University of California, San Francisco, USA; 7 Radiology, Roger Williams Medical Center, Providence, USA; 8 Russell H. Morgan Department of Radiology and Radiological Science, Johns Hopkins University, Baltimore, USA

**Keywords:** Locoregional therapy, Pancreas, Pancreatic vein, Lipiodol

## Abstract

**Purpose:**

To determine the safety and feasibility of pancreatic retrograde venous infusion (PRVI) utilizing a microvalvular infusion system (MVI) to deliver ethiodized oil (lipiodol) by means of the Pressure-Enabled Drug Delivery (PEDD) approach.

**Methods:**

Utilizing transhepatic access, mapping of the pancreatic body and head venous anatomy was performed in 10 swine. PEDD was performed by cannulation of veins in the head (*n* = 4) and body (*n* = 10) of the pancreas with a MVI (Surefire® Infusion System (SIS), Surefire Medical, Inc (DBA TriSalus™ Life Sciences)) followed by infusion with lipiodol. Sets of animals were killed either immediately (*n* = 8) or at 4 days post-PRVI (*n* = 2). All pancreata were harvested and studied with micro-CT and histology. We also performed three-dimensional volumetric/multiplanar imaging to assess the vascular distribution of lipiodol within the glands.

**Results:**

A total of 14 pancreatic veins were successfully infused with an average of 1.7 (0.5–2.0) mL of lipiodol. No notable change in serum chemistries was seen at 4 days. The signal-to-noise ratio (SNR) of lipiodol deposition was statistically increased both within the organ in target relative to non-target pancreatic tissue and compared to extra pancreatic tissue (p < 0.05). Histological evaluation demonstrated no evidence of pancreatic edema or ischemia.

**Conclusions:**

PEDD using the RVI approach for targeted pancreatic infusions is technically feasible and did not result in organ damage in this pilot animal study.

## Introduction

Pancreatic ductal adenocarcinoma (PDAC) has one of the highest cancer case fatality rates with an average 5-year overall survival (OS) of ≤ 8% for patients with advanced disease [1]. Its insidious development and aggressive biological behavior, with resistance to multimodal therapy, have made improvements in patient outcomes challenging to achieve. Combination [2] and neoadjuvant [3, 4] chemotherapy modestly improves median OS by about 8—16 weeks in advanced disease [5]. Localized delivery via intra-arterial (IA) chemotherapy, with the goal of achieving high local drug concentrations while maintaining low systemic drug level, has been reported but may be impractical in the majority of patients due to anatomical challenges [6, 7].

The highly desmoplastic architecture of PDAC leads to the relative inability of chemotherapy to penetrate the tumor even when delivered regionally via IA infusion [8] due to the presence of a dense physical barrier [9]. This same stromal architecture also results in tumors being supplied by very small feeding arteries with minimal perfusion capacity [10]. Furthermore, high solid stress and interstitial fluid pressure in excess of 130 mmHg result in the physical compression of vasculature within the PDAC tumor mass and suppression of normal convective flux of molecules from the blood stream to the tissue [11, 12]. Thus, the physical and anatomical features associated with PDAC limit the effectiveness of local IA delivery by conventional means.

To circumvent the challenges and limitations of local IA and systemic chemotherapy in PDAC, we postulate that pancreatic retrograde venous infusion (PRVI) may enable improved locoregional PDAC drug delivery, while mitigating off-target toxicity. As there are smaller arterial feeders which are often not accessible, the venous approach may overcome this limitation in pancreatic adenocarcinoma [10, 13]. We hypothesize that pressurization of the venous drainage by means of a microvalvular catheter will allow for retrograde infusion directly into the tumor vasculature. The technique, termed Pressure-Enabled Drug Delivery (PEDD), is expected to result in improved selectively, isolation, and perfusion of target tissue within the pancreas.

To test the PEDD approach, we systematically evaluated ethiodized oil (lipiodol; Guerbet, Paris, France) distribution in normal swine pancreata. The primary objective of this study was to assess the safety and feasibility of PEDD with retrograde venous infusion via microvalvular infusion catheters (MVI) and to quantify the distribution of infusate.

## Materials and Methods

### Animal Model

All animal experiments were performed under the supervision and with approval of the T3 Research Institute Animal Care and Use Committee (AAALAC accredited). We tested healthy, growing swine (50–60 kg, *n* = 10). All were sedated with an intramuscular injection of a mixture of ketamine (22 mg/kg), acepromazine (1.1 mg/kg), and atropine (0.05 mg/kg). A single dose of antibiotics, Dual-Cillin (300,000U/mL, i.m.), was administered prior to the interventional procedures. Intravenous pentobarbital (20 mg/kg body wt) was used to induce the animal after which the animals were intubated and mechanically ventilated with 2% isoflurane and 98% oxygen.

### Pressure-Enabled Drug Delivery (PEDD) with Microvalvular Infusion System

The Surefire® Infusion System (SIS) (Surefire Medical, Inc (DBA TriSalus™ Life Sciences), Westminster, CO) is a 3.4F coaxial infusion microcatheter having a 0.025″ inner lumen with a microvalve at the distal end that serves as the conduit for physician-specified agents. The distal funnel-shaped microvalve is manufactured in two sizes, covering vessels ranging from 2–4.0 mm (025 M configuration) and 4.0–6.0 mm (025L configuration).

### Pancreatic Venous Anatomy Evaluation (Figs. 1 and 2)

**Fig. 1:**
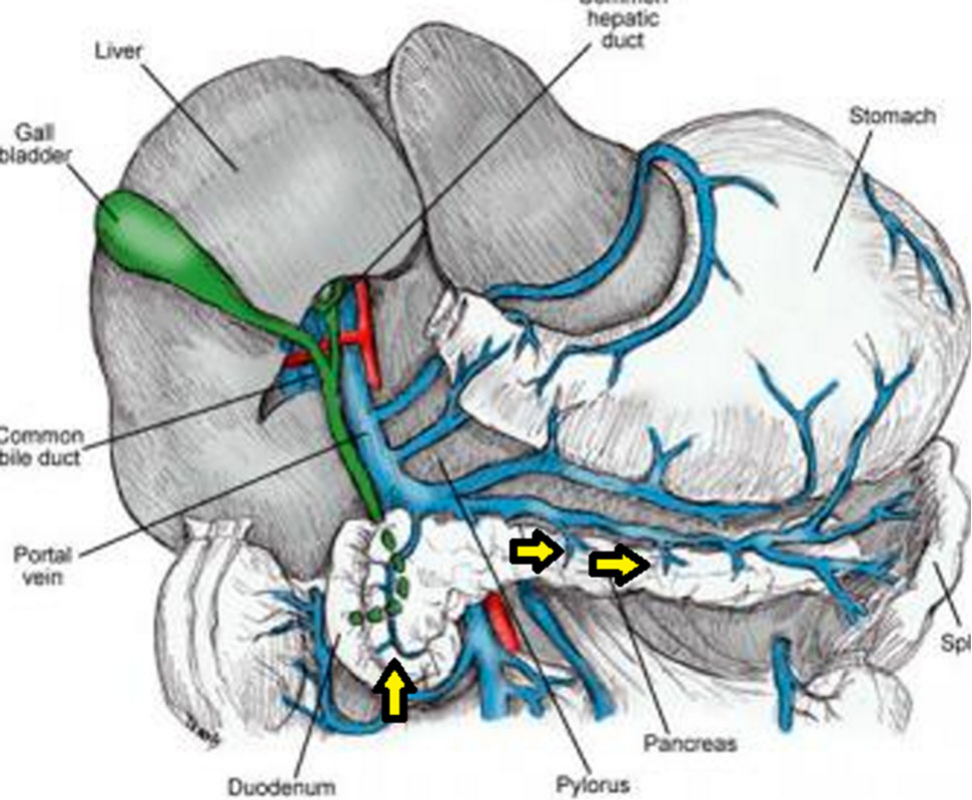
Artistic rendering of pancreatic venous anatomy (Image reproduced with permission from Medscape Drugs & Diseases (https://emedicine.medscape.com/), Pancreas Anatomy, 2017, available at: .) Yellow arrow = pancreatic veins

**Fig. 2:**
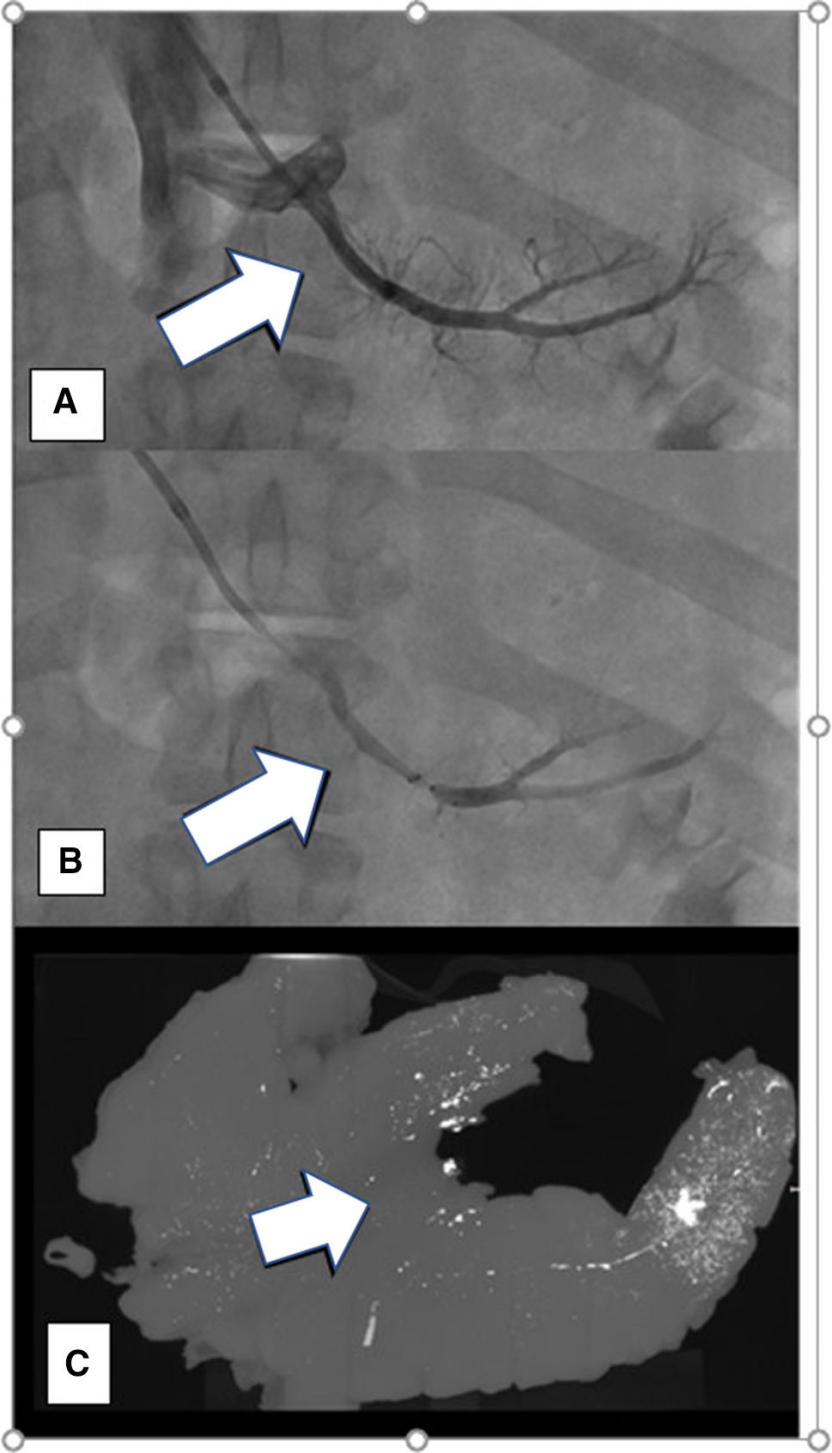
Transhepatic venous delivery into the pancreatic veins draining the body/tail (White arrow = body) . **A** Contrast venography (anteroposterior view) of the pancreatic vein draining the pancreatic body (white arrow). Pancreatic vein is arising off the splenic vein. **B** Placement of microvalvular system (white arrow) into the pancreatic vein to isolate the pancreatic body. **C** Ex vivo three-dimensional volumetric coronal MIP (micro-CT) of the pancreas after lipiodol injection. A circular region of interest (ROI) was placed in the area of maximum density (yellow circle) and in background tissue (white arrow) to calculate SNR

The study was performed in ten swine. Angiography was performed on a Phillips system, Integra Flat Panel. Percutaneous access to the portal vein was achieved using ultrasound guidance with a transhepatic technique. A 22-gauge Chiba needle (Neff Percutaneous Access Set; Cook, Bloomington, Ind) was placed into a branch of the right portal venous system. After the portal vein was accessed, a 5-French sheath (Cordis, Miami, FL) was placed in the main portal vein. Flush portography was performed with a 5-F angiographic pigtail catheter placed in the splenic vein. Using standard 5 French angiographic catheters (Omni Sos, Angiodynamics, Queensbury, NY), mapping of the pancreatic venous anatomy was performed to identify the venous drainage of the pancreatic body/head and to assess for collateral venous drainage. Venous collaterals, when present, were embolized using coils (6–8 mm Nester Embolization Coils; Cook, Bloomington, Ind) and gelfoam to limit non-targeted delivery of lipiodol. Next, cannulation of the main pancreatic venous drainage of the head (*n* = 4) and body (*n* = 10) was performed with the MVI system. Subselective venograms were performed after devices were deployed in the head or body to confirm appropriate positioning. Next, second-order branches were selectively catheterized utilizing diagnostic catheters and the MVI systems with 0.016″ Fathom guidewires (Boston Scientific, Marlborough, MA). Pancreatic vein diameters were measured in the main and second-order branches, and flow dynamics were assessed in the distal vascular bed after the MVI systems were deployed.

A total of 14 PRVIs were performed in a total of 10 swine (pancreatic head = 4 and body = 10). The endpoint of embolization was of stasis or sluggish flow with leakage of lipiodol through the MVI system. After infusion of ethiodized oil (lipiodol; Guerbet, Paris, France), catheters were flushed with a 5 cc of saline to clear any residue at the tip. After the procedure, all catheters and sheaths were removed. In the survival animals (*n* = 2), the access to the portal vein was closed by a transhepatic deployment of a Mynx Vascular Closure Device (AccessClosure, Inc, Santa Clara, CA). Two animals were killed 4 days post-RPVI. Eight animals were killed immediately post-RPVI. All animals were humanely euthanized by an IV injection of barbiturates (e.g., ~ 100 mg/kg pentobarbital) while under general anesthesia followed by potassium chloride to arrest the heart in end diastole. All pancreata were then harvested for analysis.

### Biochemical Analysis

Two swine underwent standard laboratory assessment pre-procedure, post-procedure, 24 hr, and 96 hr (4 days) post-infusion prior to explant. This included standard comprehensive metabolic panel, complete blood count, and amylase analysis.

### Ex Vivo Lipiodol Distribution Analysis

After the infusion procedure, high-resolution images of the explanted pancreata were analyzed for lipiodol distribution pattern, clumping, and extension of lipiodol into non-target regions in the pancreas, duodenum, and gallbladder. All explanted pancreata and duodenums underwent micro-CT imaging (GE eXplore CT 120™ System, Milwaukee, WI). 3D volumetric/multiplanar MIP images of were performed utilizing an Osirix Workstation (Osirix 3.8.1, 64 bit, Geneva, Switzerland). Data sets were generated for each pancreas (*n* = 10) and explanted duodenum and gallbladder (whole coronal plane, whole sagittal plane, whole axial plane) for 3D volume-rendered MIP and multiplanar reconstructions (MPR) images with 1-mm slice thickness (approximately 25 slices per pancreas). To obtain attenuation values of the tissue, each slice of the micro-CT image was aligned to the midline of each pancreas, and a region of interest was placed in the area of maximum density. Ten regions of interest were generated in the body and head of the pancreas in the areas of infusion. To achieve consistency, a circular region of interest (ROI) was used, and the size was adapted to analyze a 4-mm^2^ portion of the pancreas. In a similar manner, circular ROIs were used in extra pancreatic tissue (explanted gallbladder and duodenum). Quantitative assessment of the signal-to-noise ratio (SNR) in various parts of the pancreas (head, body/tail) and extra pancreatic tissue was measured using circular ROIs. The ROI and standard deviation (SD) for the background were also obtained. The SNR values for the pancreatic head, tail, and extra pancreatic tissue (duodenum and gallbladder images) were calculated using the mean pixel intensity (I) of the ROI in the target tissue divided by the SD of background, the “estimated noise.”

$${\text{SNR }}\left( {{\text{ROI}}} \right){\text{ } = \text{ Mean I }}\left( {{\text{ROI}}} \right) \div {\text{SD I }}\left( {{\text{noise}}} \right)$$

Statistical analysis was performed using an unpaired t-test to compare the SNR of the pancreatic head (target) to tail (non-target) and the SNR of pancreatic tissue to extra pancreatic tissue. Analysis was conducted using GraphPad Prism (GraphPad Software, San Diego, California).

### Histopathological Analysis

Several one-gram tissue samples were collected from the infusion region within the pancreas and from the other regions of interest in the gallbladder and duodenum adjacent to the infusion site. The tissues were then fixed in 10% neutral-buffered formalin. The fixed samples were then sent to HistoWiz (Brooklyn, NY) for staining and analysis. Histology was performed by HistoWiz according to Standard Operating Procedures. Submitted tissues were sectioned ~ 10 um thick and stained for hematoxylin and eosin (H&E) histology stain. Whole slide scanning (40X) was performed on an Aperio AT2 (Leica Biosystems).

Microscopic evaluation and morphometry were performed by trained personnel. Scanned slide images were utilized for microscopic evaluation and photomicrographs.

## Results

### Pancreatic Venous Anatomy Evaluation (Figs. 1, 2 and 3)

**Fig. 3:**
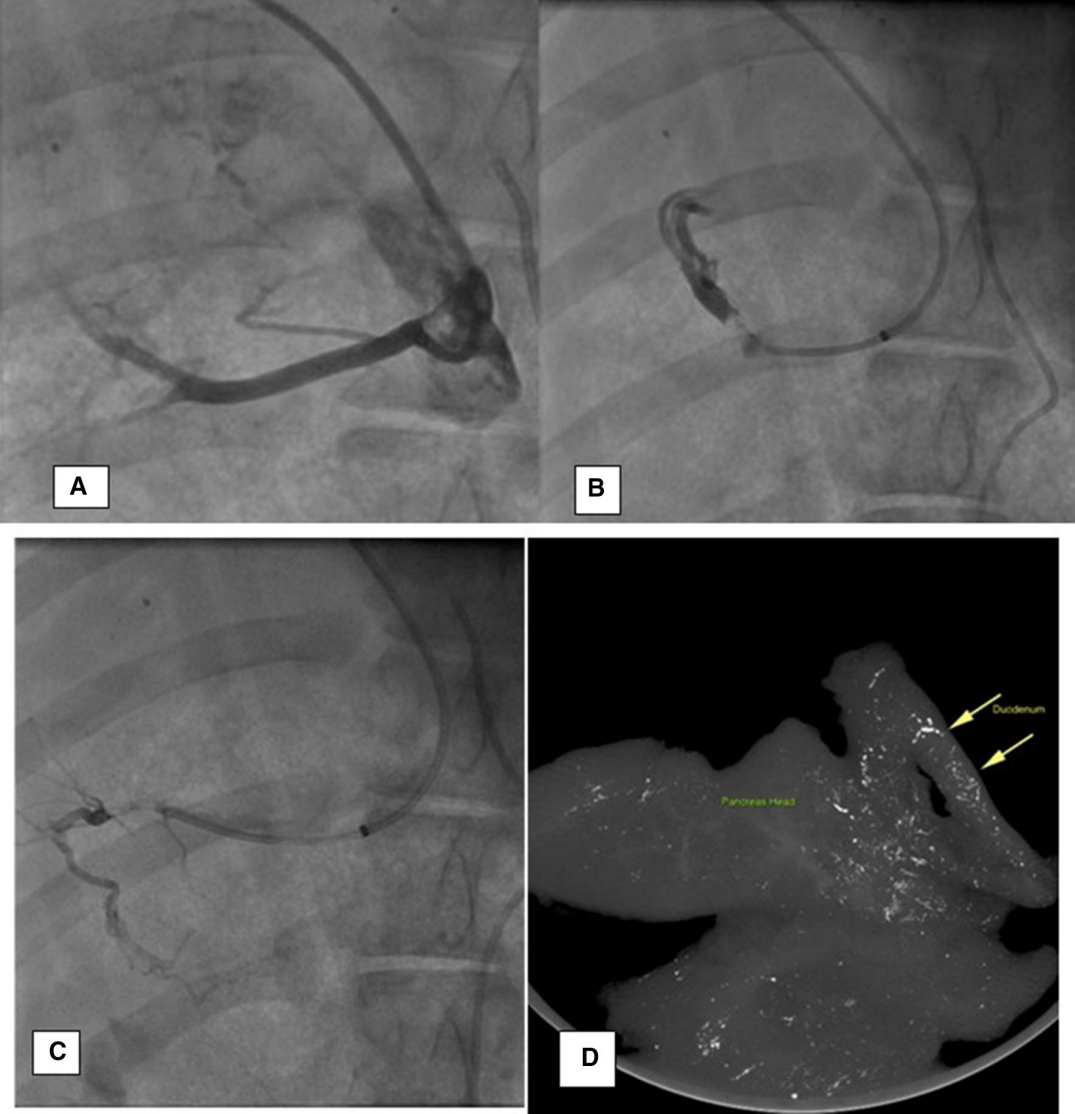
Transhepatic venous delivery into the pancreatic veins draining the head. **A** Contrast venography (anteroposterior view) of the superior pancreato-duodenal branch off the portal vein. **B** Cannulation of duodenal branch with microvalvular system prior to embolization with gelfoam. **C** Cannulation of pancreatic head vein with microvalvular system. **D** Ex vivo three-dimensional volumetric coronal MIP (micro-CT) of pancreatic head and duodenum

A total of 14 PEDD procedures were conducted via PRVI in a total of ten swine without any complications. Due to the presences of collaterals arising off the inferior pancreaticoduodenal branches, embolization of these vessels was necessary to target pancreatic head infusions. With pancreatic body infusions, the main pancreatic vein draining into the splenic vein was utilized, with no significant collaterals detected.

In 4 swine, angiography revealed vasospasm or small vascular perforations in the side branch vessels related to guidewire manipulation which did limit therapeutic delivery. An average of 1.7 mL (range 0.5–2.0 mL) of lipiodol was infused in each target. Micro-CT provided high-resolution visualization of the pancreatic parenchyma with 70 µm isotropic resolution. Lipiodol deposition was present in the deep pancreatic parenchyma as confirmed by micro-CT but not always readily visible under fluoroscopy.

### Ex Vivo Qualitative Analysis

3D MIP and 3D MPR images generated from micro-CT data sets showed the distribution of lipiodol predominately in the target tissue bed of the pancreas body or tail (Figs. 2C and 3D). In contrast, PRVI into the pancreatic body demonstrated accumulation of lipiodol in the pancreatic tail and head suggestive of intra-pancreatic collaterals which were not readily visible at the time of procedure. PRVI delivery of lipiodol to the pancreatic head resulted in a low degree of non-target accumulation present in the duodenum and gallbladder (Fig. 3D). Qualitative examination of the target pancreatic vascular bed showed a distribution of the lipiodol throughout the parenchyma with extension into the deeper tissue parenchyma.

### Biochemical Analysis (Table 1)

**Table 1:** Laboratory Values Pre- and Post-Treatment at 5 Days

Parameter	Reference	Unit	Animal 1				Animal 2			
			Pre-procedure	Post-procedure	24 h	96 h	Pre-procedure	Post-procedure	24 h	96 h
Total protein	5.0–7.0	g/dL	5.8	5.1	5.5	5.5	6.2	6.2	6.5	6.2
Albumin	2.4–3.3	g/dL	3.7	3.2	3.6	3.4	3.6	3.5	3.9	3.6
AST (SGOT)	10–100	IU/L	22	22	55	90	24	26	34	28
ALT (SGPT)	10–100	IU/L	31	26	30	46	26	25	27	36
Alk phosphatase	100–250	IU/L	233	231	225	187	115	116	122	115
GGT		IU/L	38	43	41	39	30	28	32	37
Total bilirubin	0.0–1.0	mg/dL	0.3	0.5	0.1	0.1	0.2	0.3	0.1	0.1
Phosphorus	3.0–5.5	mg/dL	9.1	9.6	7.2	8.1	8.1	7.6	6.9	8.1
Glucose	70–120	mg/dL	85	70	102	91	83	86	95	94
Calcium	7.2–11.5	mg/dL	10.3	9.7	10	10.2	10.2	10.5	10	10.4
Magnesium		mEq/L	1.5	1.5	1.7	1.7	1.4	1.2	1.8	1.7
Amylase	1000–2500	IU/L	2,186	1,925	2,030	1,908	2,396	2,315	2,137	1,637
CPK	100–400	IU/L	3,988	3,541	6,123	7,108	1,033	954	1,612	541
LDH		IU/L	426	371	601	1,137	367	342	457	439
WBC	9.6–25.2	10^3/uL	15.4	15.1	13.9	16	13.2	14.2	12.1	12.4
RBC	4.9–7.9	10^6/uL	6.4	6	6.7	5.9	6.6	6.5	6.9	6.5
HGB	8.1–11.9	g/dL	11.6	10.9	12.2	10.5	11.2	11	11.8	10.9
HCT	28–40	%	36	34	40	35	35	35	39	37
Platelet count	200–800	10^3/uL	214	251	229	137	266	233	449	376

*ALT* alanine aminotransferase, *GGT* gamma glutamyltransferase, *AST* aspartate aminotransferase, *INR* international normalized ratio, *CPK* creatinine phosphokinase, *WBC* white blood count, *RBC* red blood cell count, *HGB* Hemoglobin HCT = hematocrit.

Laboratory values from 2 animals did not display notable changes from pre-procedure baselines at the post-procedure, 24-hr, and 96-hr time points. High albumin, phosphorus, and creatinine phosphokinase were noted pre-procedure and persisted throughout the duration of the study. All other indicators remained within normal ranges after PRVI.

### Ex Vivo Quantitative Analysis (Figs. 4 and 5)

**Fig. 4:**
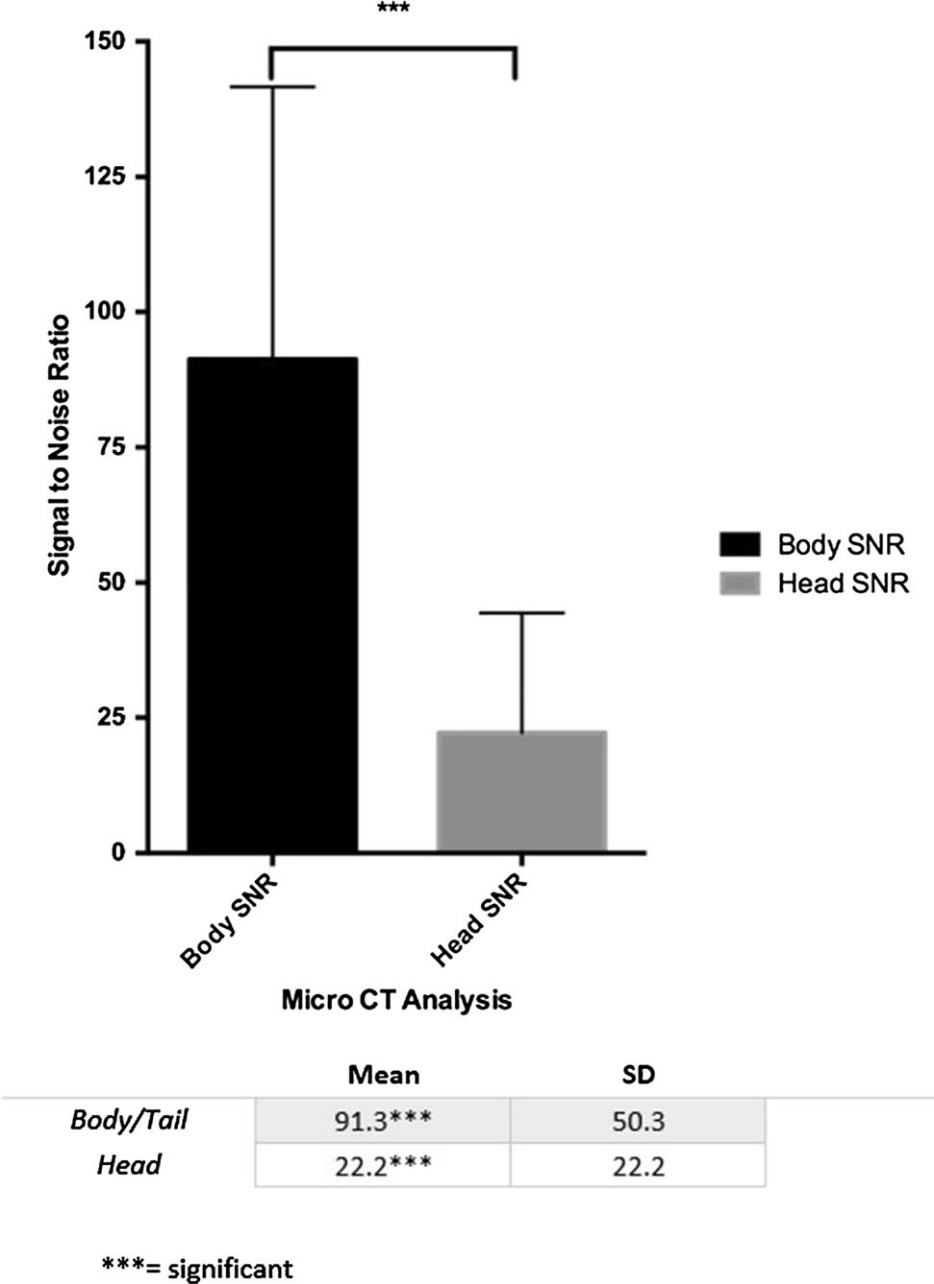
Quantitative assessment of lipiodol distribution utilizing SNR in the pancreatic body vs head based on micro-CT analysis

**Fig. 5:**
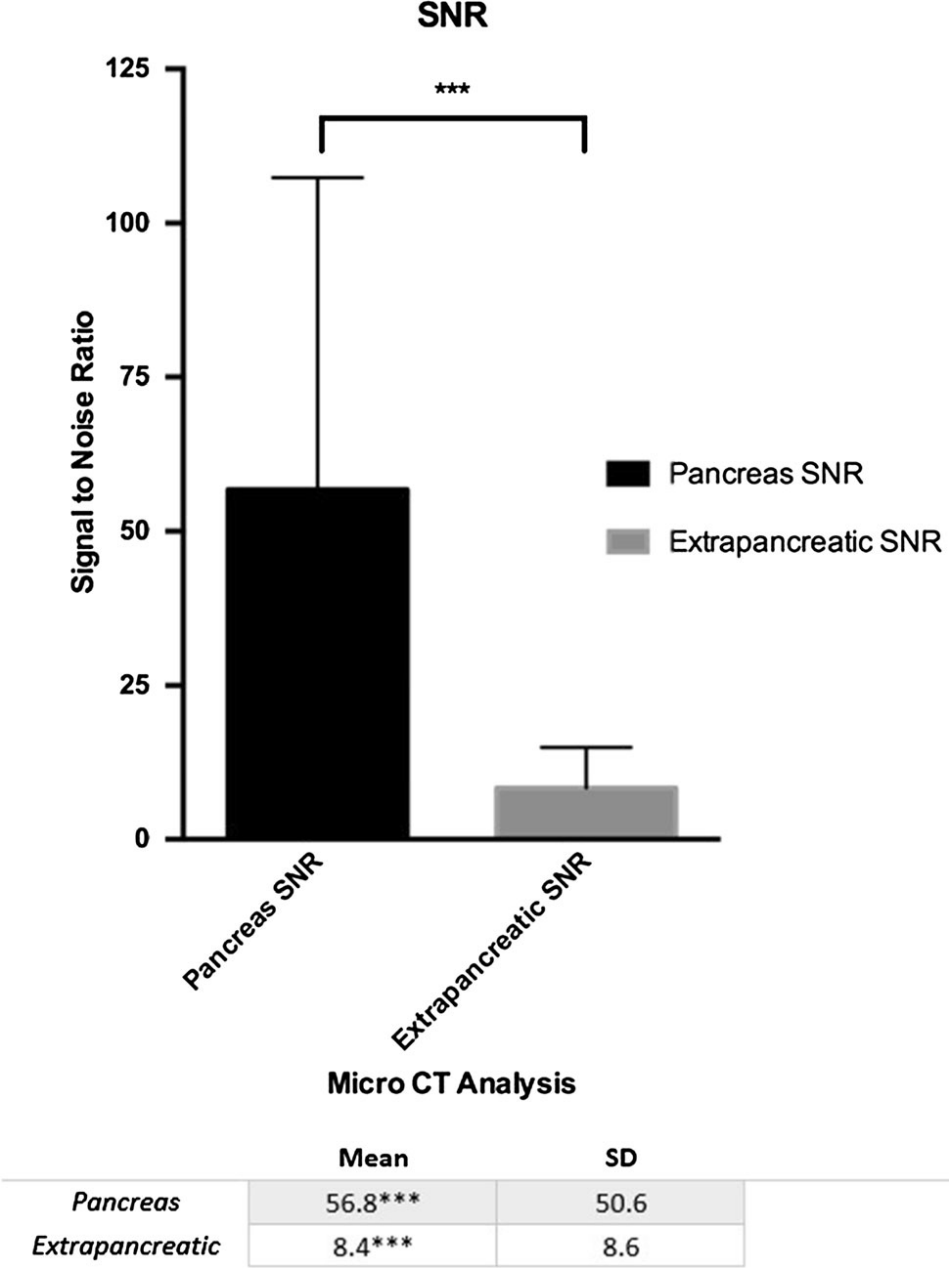
Quantitative assessment of lipiodol distribution utilizing SNR in the pancreas vs. extra pancreatic tissue based on micro-CT analysis

SNRs of lipiodol deposition was increased in the pancreatic body/tail in comparison with the pancreatic head (*p* = 0.009). The SNR of the pancreatic tissue was shown to be significantly higher in the pancreas versus extra pancreatic tissue (gallbladder and duodenum, *p* = 0.0001).

### Histopathological Analysis (Fig. 6)

**Fig. 6:**
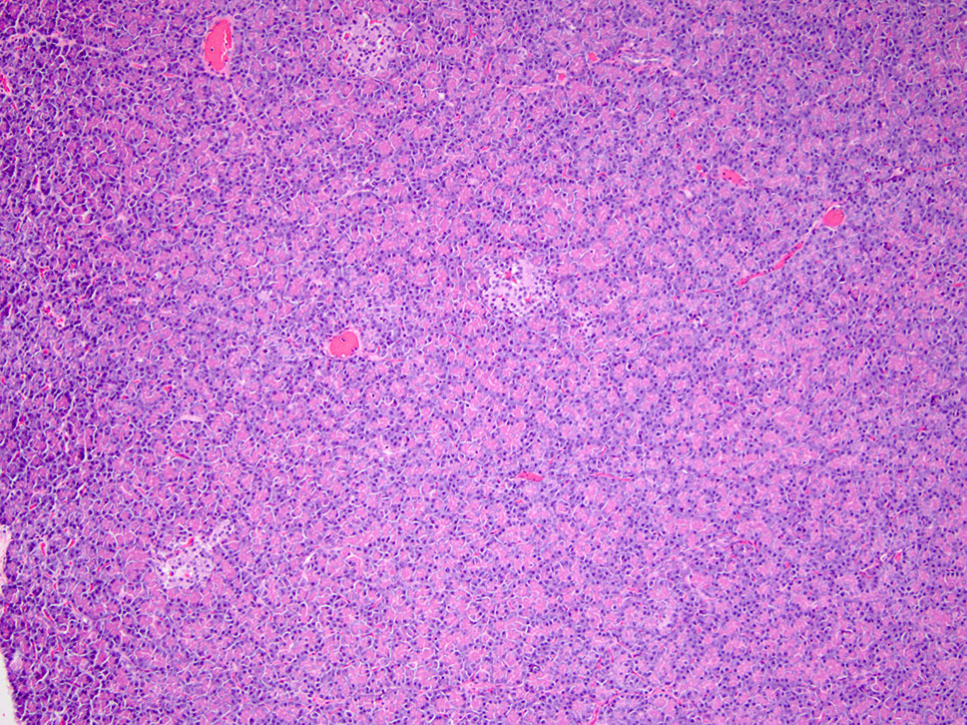
Hematoxylin and eosin (H&E) histology of the pancreatic body (infusion site); microscopically evaluated thin sections of the pancreas show no evidence of necrosis, fibrosis, or inflammation

Pancreatic tissue architecture was intact without any significant inflammation, ischemia, edema, or tissue damage.

## Discussion

The main finding of our study is that the PEDD approach in the pancreatic venous system is technically feasible in a large animal model. Using a combination of radiopaque lipiodol, a microvalvular infusion system, and 3D volumetric imaging with micro-CT, we confirmed that PRVI of agents into the pancreatic tissues is achievable. In addition, based on SNR calculations, there was a statistically significant increased amount of lipiodol in the targeted pancreatic body versus the pancreatic head and similarly versus extra pancreatic tissue. Thus, PEDD may serve as a viable alternative for locoregional delivery of therapeutics into pancreatic tissue.

All prior vascular efforts targeting pancreatic cancer have utilized an arterial approach via the celiac and/or splenic arteries [14, 15]. As the feeding arteries directly into the tumor are often too small to catheterize, the current IA strategy is to flood and saturate the arterial system closest to the tumor bed [16]. The IA methodology requires extensive catheterization and embolization of multiple vessels in order to “skeletonize” the arterial system so the therapeutic agent only travels in one of the main arteries (celiac, gastroduodenal or splenic artery) adjacent to the tumor [5, 6, 16]. Furthermore, as the systemic exposure is significant, these patients often undergo multiple repeated procedures with lower doses in order to achieve a sufficient therapeutic index [17].

Nevertheless, clinical trials have shown that regional IA infusion with gemcitabine may have the potential to improve the response and resectability rates for advanced pancreatic cancer in highly selected patients. A meta-analysis of 298 patients (both stage 3 and 4) undergoing IA delivery using gemcitabine showed a survival benefit of 30% at 1 year when compared with systemic gemcitabine [6]. In addition, Rosemurgy and colleagues reported a study in which 20 subjects with locally advanced PDAC received 100 catheter-directed IA infusions, with the number of procedures reflective of the technical challenges. In 15 evaluable patients, the 1-year survival rate was 60% and 58% of the population had CA19-9 reductions [5]. However, all these studies are significantly hampered by serious adverse events which include sepsis, arterial dissection, hyperglycemia, and neutropenia which are usually attributable to non-target delivery [5]. In addition, the studies did not contain control arms and likely reflect a high degree of selection bias, while the follow-up times were too short to draw meaningful outcome conclusions.

Arterial infusion methods are fundamentally limited because pancreatic tumors have poor arterial perfusion compared to surrounding healthy tissue due to the desmoplastic stroma [18, 19]. Thus, flow directed therapies such as IA infusions often lead to non-target delivery. Despite the use of extensive embolization techniques to isolate the vasculature, the inability to target the tumors remains significant [7, 16]. Furthermore, hypoxia and decreased vascularity in combination with elevated interstitial pressures prevent chemotherapeutic agents from entering the tumor [15, 20].

Our pilot results suggest the alternative transvenous route of delivering chemotherapy to PDAC, coupled with PEDD, may overcome some of the limitations associated with the IA approach. In comparison with systemic intravenous and locoregional IA therapies, PEDD in the venous system does not rely on the limited arterial supply and instead takes advantage of the more easily accessible venous branches which drain the pancreatic tumor [13].

Although this is a unique approach for the pancreas, this methodology has been utilized in other organ systems where there is limited arterial access. For example, in the setting of cellular/gene therapies, retrograde transvenous methodology has been described in the targeting of myocardium and extremity musculature associated with coronary artery disease and muscular dystrophy, respectively [21–23]. In this technique, therapeutics are delivered in a retrograde fashion directly into the myocardium or target extremity via the coronary sinus or the main venous drainage of the affected extremity. Thus, there is a growing awareness on utilizing this approach for locoregional therapies.

Utilizing 3D volumetric micro-CT imaging analysis (isotropic resolution of 70um) and the mathematical calculation of SNR, we were able to accurately depict and quantify deposition of lipiodol in various parts of the pancreas. In our study, the SNR in the pancreatic body was 91.3 (+ /50.3) compared to the non-target pancreatic head, which was 22.2 (± 22.2). Although SNR calculations with lipiodol are a crude surrogate for tissue-level concentrations, it appears that highly concentrated and targeted delivery is achievable. Although we did notice delivery of lipiodol in extra pancreatic tissue such as duodenum and gallbladder during pancreatic head infusions, the delivery to the pancreatic tissue was still significantly higher (*p* = 0.0001). Of note, we did not notice any lipiodol within the gastric mucosa. Lastly, the imaging data and biochemical analysis at 4 days suggests the targeting via PEDD is both durable in terms of therapy remaining in the target tissue and is a strong indicator that the method successfully deposited therapy into regions of poorly perfused tissue.

## Limitations

Our study has several limitations. First, only two animals of the 10 underwent a 4-day survival. Therefore, the long-term in vivo effects of this method have only undergone a limited assessment with this investigation. Further analysis would require a longer-term survival with comparison of transvenous delivery to systemic chemotherapeutic regimens serving as controls. In addition, a refined quantitative estimation of the distribution of radiopaque lipiodol in a tumor model was not performed. The opportunity to test this type of technique in an oncology setting would be of interest and provide important data both on infusion and locoregional response to therapy. However, a workable porcine model of pancreatic cancer, termed Oncopig, is undergoing development/validation and may be an option for future studies [24]. Finally, we speculate that there may be some technical challenges that need to be further elucidated in the clinical setting. As shown in our study, this technique may have the potential for non-target delivery, particularly in inoperable neoplasms. As PDAC tumors are known to invade mesenteric vasculature, retrograde infusion could potentially deliver a therapeutic to draining viscera such as small bowel. Thus, in these types of tumors, a more detailed analysis of the involved visceral vessels needs to be well understood prior to the procedure. For the time being, we intend to exclude patients with mesenteric or portal venous occlusion. Finally, should this technique may require multiple treatments, the added risk of repeated portal venous punctures must be weighed in designing therapeutic plans [24].

## Conclusion

In summary, PRVI utilizing the PEDD approach is technically feasible and associated with no significant organ damage in this pilot animal study. The ability to deliver therapy selectively into the pancreatic head and body is possible with improved targeting to the pancreatic body.

## Abbreviations

ALT: Alanine aminotransferase
MVI: Microvalve infusion catheter
AST: Aspartate aminotransferase
IRE: Irreversible electroporation
PRVI: Pancreatic retrograde venous infusion
